# Purification and characterization of bacteriocin produced by a strain of *Lacticaseibacillus rhamnosus* ZFM216

**DOI:** 10.3389/fmicb.2022.1050807

**Published:** 2022-11-10

**Authors:** Danli Wu, Mengdi Dai, Yongqing Shi, Qingqing Zhou, Ping Li, Qing Gu

**Affiliations:** Key Laboratory for Food Microbial Technology of Zhejiang Province, College of Food Science and Biotechnology, Zhejiang Gongshang University, Hangzhou, China

**Keywords:** *Lacticaseibacillus rhamnosus* ZFM216, purification, bacteriocin ZFM216, characteristics, mode of antibacterial

## Abstract

The recent surge in demand for natural preservatives has ushered in a new era of research into novel bacteriocins capable of effectively combating food-borne infections. In this study, the bacteriocin from *Lacticaseibacillus rhamnosus* ZFM216, which has a molecular mass of 11851.9 Da, was purified using macroporous resin, gel chromatography, and reversed-phase high performance liquid chromatography. This bacteriocin could inhibit both Gram-positive and Gram-negative bacteria. It had a strong inhibitory effect on *Staphylococcus aureus* D48 with minimum inhibitory concentration values of 1.75 μM. Bacteriocin ZFM216 was heat stable and showed pH stability under weakly acidic conditions. It was sensitive to pepsin, proteinase K and trypsin. Electron microscopy results showed that when treated with bacteriocin ZFM216, *S. aureus* D48 was severely deformed, the cell structure was obviously changed, and the intracellular electrolyte leaked to the outside of the cell. Bacteriocin ZFM216 caused the ATP level of the indicator to decrease, the conductivity to sharply increase, and the transmembrane potential difference (ΔΨ) to instantaneously decrease. This research formed the basis for further development and utilization of bacteriocin ZFM216 which has potential in the food industry.

## Introduction

Microbial food safety is a globally severe concern (Luo et al., 2022). It is related to a large number of diseases that harm our society and economy (Bintsis, 2017; Liu et al., 2022). Food spoilage bacteria are the main cause of food spoilage. Chemical preservatives are usually used to prevent the microbial pollution of food, which is harmful to human health to a certain degree. Therefore, it is extremely necessary to seek native compounds’ bioactivities which have potential applications in food control (Luo et al., 2022).

Bacteriocin is a safe and effective antibacterial peptide synthesized by ribosomes during bacterial metabolism. Bacteriocins were roughly classified into four classes. Class I bacteriocins, known as lantibiotic, are posttranslationally modified protein with the molecular weight of <5 kDa. Class I bacteriocins is further subdivided into class Ia (lantibiotics), class Ib (labyrinthopeptins) and class Ic (sanctibiotics). Nisin is a most popular class Ia bacteriocin (Breukink, 1999). Class II bacteriocins, nonlantibiotics, are thermostable, which can be further sub-divided into class IIa (pediocin-like bacteriocins, with N-terminal conservative sequence (YGNGV)), class IIb (two-peptides unmodified bacteriocins), class IIc (circular bacteriocins) and class IId (unmodified, linear, non-pediocin-like bacteriocins; Kumariya et al., 2019). Class III bacteriocins are nonlantibiotics with heat sensitivity, and its molecular weight generally >30 kDa. Colicin is a Class III bacteriocin produced by *Escherichia coli*. Class IV bacteriocins are comprised of lipid or carbohydrate groups (Cui et al., 2021). Bacteriocins has great potential in preserving foods such as fruits, vegetables, meat and dairy products (Cui et al., 2021). Bacteriocins synthesized by lactic acid bacteria (LAB) can be developed as novel alternative biopreservatives and food preservation strategies (Kaškonienė et al., 2017; Luo et al., 2022). For example, nisin is used for antisepsis and preservation in the food industry (Rashid et al., 2016). Pediocin, which has been permitted to enter the market as Alta™ 2341 and Microgard™ (Breukink, 1999), can inhibit *Listeria monocytogenes* in meat and dairy products. However, these commercial bacteriocins are limited by their narrow antibacterial spectrum, so the development of new bacteriocins that could efficiently inhibit more food-borne pathogens is essential. Efficient purification of bacteriocins has always been a concern of researchers.

Depending on their solubility, bacteriocins can be separated using ammonium sulfate precipitation (Yi et al., 2016) and organic solvent extraction (Zhao et al., 2016). Plantaricin K25 was purified by (pH-mediated) cell adsorption and desorption (Wen et al., 2016). In addition, membrane filtration is also a normal method to obtain purified bacteriocin (Abanoz and Kunduhoglu, 2018). According to molecular sieving and adsorption, plantaricin ZJ008 was obtained using macroporous resin XAD-2 (Zhu et al., 2014). Ion exchange chromatography can also be used to separate bacteriocins due to their different charges (Dündar, 2016). Based on molecular weight, Chang (Chang et al., 2013) used gel chromatography to isolate the enterocin TW21 from *Enterococcus faecalis*. Li (Yi et al., 2016) isolated a new bacteriocin from *Lactobacillus crustonum* MN047 by high performance liquid chromatography (HPLC). Ye et al. (2021) obtained bacteriocin ZFM54 from *Lacticaseibacillus paracasei* ZFM54 using macroporous resin, cation-exchange chromatography, Sephadex gel filtration and HPLC. Generally, separation and purification operations are time-consuming and not suitable for all samples (Cui et al., 2021). Therefore, it is crucial to develop efficient and targeted methods for specific bacteriocin purification.

Some bacteriocins kill bacteria by damaging their cell membranes (Liang et al., 2022). For example, nisin is capable of binding with lipid II to interrupt the production cycle of the multienzyme peptidoglycan, thereby blocking cell wall synthesis (Medeiros-Silva et al., 2019). In addition, nisin can also form transmembrane ion channels on the membrane of target bacteria, leading to the leakage of cell contents and subsequent killing of the bacteria (Grein et al., 2019). Some bacteriocins can enter the target cell and affect its metabolism (Brogden, 2005). Lantibiotic 481 can prevent peptidoglycan from synthesizing the cell wall (Knerr and van der Donk, 2012); Pyrrhocoricin kills bacteria by inhibiting the translation process in protein synthesis (Taniguchi et al., 2016); Bacteriocin F1(Miao et al., 2016) and buforin II (Park et al., 1998) can bind to the DNA of target bacteria and cause cell death. Although many antibacterial bacteriocins have been discovered worldwide, the antibacterial modes of most bacteriocins have not been clarified, which seriously limits their industrial application. Focusing on the mechanism of bacteriocins can help provide a theoretical foundation for the application of novel bacteriocins (Muhammadar et al., 2018).

A three-step method was used in this research to purify the bacteriocin secreted by *L. rhamnosus* ZFM216. During this research, the stability of this bacteriocin and its antibacterial mode-of-action were also investigated to provide a theoretical basis for its application in food protection.

## Materials and methods

### Source and culture conditions of strain

*L. rhamnosus* ZFM216 was grown in de Man Rogosa and Sharpe (MRS) broth at 37°C for 24 h and stored at −80°C. It was isolated from raw milk, Hangzhou dairy farm in Zhejiang Province, China. This strain is now kept in the China Center for Type Culture Collection (CCTCC; Wuhan, China) with the identifier of CCTCC M 2020325. The strains used for the antibacterial spectrum were grown in LB broth for 24 h at 37°C.

### Purification of bacteriocin

This bacteriocin was produced using a three-step method, including the using of macroporous resin XAD-16 column (H&E, China), dextran gel chromatography with a Sephadex™ LH-20 column and reversed-phase HPLC (RP-HPLC) with a SunFire™ Prep C18 column (10 × 100 mm). The *L. rhamnosus* ZFM216 strain was cultivated with the MRS liquid medium under 37°C for 24 h. The inoculation size was 2% (v/v) and the fermentation volume was 1 L. The bacterial suspension was centrifuged at 8,500 rpm and 4°C for 30 min using an ultrahigh-speed centrifuge (Beckman, American). The cell-free supernatant (CFS) was collected, and the precipitated bacteria were discarded. The CFS was first passed through the XAD-16 column at a flow rate of 2 mL/min and washed with 2 L ultrapure water. Then, this column was washed with 500 mL of 20%, 30%, 40%, 60%, 80% and 100% ethanol at the same flow rate. The eluent was collected under each gradient and concentrated to 50 mL by rotary evaporation. Then the antibacterial activity of each gradient eluent was detected by the Oxford cup agar diffusion method, where *S. aureus* D48 was selected as the indicator. The antimicrobial activity part was collected, and Sephadex LH-20 gel chromatography was carried out for the next step. Ultrapure water was used for equilibration and elution at 1.2 mL/min. The eluant was collected every 3 min using separate tubes each time. The collected samples were detected by spectrophotometry (Waters, United States) at 280 nm and 215 nm to generate the absorption curves. The antibacterial activities of the samples at the absorption peaks were tested by the Oxford cup method. Next, the activity peaks from gel chromatography were collected and further separated by RP-HPLC (Waters 2998, United States). A Sunfire™ Prep C18 column (5 μm, 10 × 100 mm) was utilized. After passing through the water filter membrane (0.22 μM), the sample settled at 4°C. The injection volume was 5 mL, buffer A was ultrapure water (0.05% v/v TFA), and buffer B was acetonitrile (0.05% v/v TFA). Gradient elution ranged from 95% buffer A to 95% buffer B, with a flow rate of 4.0 mL/min. In this research, each single peak was collected, and antibacterial activity was determined after removing the organic solvent by evaporation. After freeze-drying, the above active components were redissolved with 1 mL of ultrapure water and confirmed by analytical HPLC (Waters 2998 HPLC, United States). The chromatographic column was a Sunfire™ prep C18 (5 μm, 4.6 × 250 mm). The injection volume was 30 μL, and the buffers were the same as those previously mentioned. The protein content was measured using a bicinchoninic acid (BCA) protein quantification kit (Takara, China).

### Determination of the molecular weight of bacteriocin

After purification, Tricine-SDS-PAGE was used to roughly determine the molecular mass. Matrix-assisted laser desorption time-of-flight mass spectrometry (MALDI-TOF-MS) was used to confirm the molecular weight of this bacteriocin. The sample was mixed 1:1 with matrix containing α-cyano-4-hydroxycinnamic acid (1:1) and detected using a 355 nm Nd-YAG laser with a cyclotron frequency of 400 Hz, accelerating voltage of 20 kV and positive ion mode.

### Antibacterial spectrum and minimum inhibitory concentration

The agar-well diffusion method was used for determining the bacteriostatic spectrum. Every 50 μL, 0.5 mg/mL bacteriocin was added to the wells of each plate with 10^6^ CFU/mL of different indicator strains. After overnight cultivations at 37°C, the diameter of the bacteriostatic circle was measured.

The MIC for different indicator strains was tested using the 96-well plate method. The concentration gradient of bacteriocin was 1.28, 0.64, 0.32, 0.16, 0.08, 0.04, 0.02, 0.01, 0.005 and 0 mg/mL. Absorbance at OD_600_ was measured by an ultraviolet spectrophotometer (OLYMPUS, Japan).

### Stability against heat, pH, and enzyme

For temperature stability evaluation, every 200 μL of 0.4 mg/mL bacteriocin ZFM216 was heated to 50, 60, 70, 80, 90 and 100°C for 1 h and then cooled them to 25°C. The relative bacteriostatic activity of bacteriocin ZFM216 was measured at each temperature. Unheated bacteriocin ZFM216 was used as control (CK).

For pH stability evaluation, the pH value of purified bacteriocin ZFM216 was changed to 3–9 by using 1 M NaOH or 1 M HCl and maintained at the corresponding pH for 20 min. The final concentration of bacteriocin was 0.4 mg/mL. The above relative bacteriostatic activity of bacteriocin ZFM216 were measured at each pH. The highest value was set as baseline (100%).

For proteolytic enzyme treatments, papain (pH 7.0), pepsin (pH 3.0), trypsin (pH 8.0) and protease K (pH 3.0) were prepared into 200 μL 2 mg/ml solutions, and bacteriocin was added in each system. The mixed solution with a final concentration of bacteriocin of 0.4 mg/ml was incubated for 1 h at 37°C and then placed into a water bath at 100°C for 5 min to inactivate the enzyme. The pH of the mixture was adjusted to its original level. The untreated bacteriocin was used as control (CK).

*S. aureus* D48 was used to determine the antimicrobial activity using the agar-well diffusion test. And the relative bacteriostatic activity were calculated. The experiments were performed in triplicate.

### Scanning electron microscopy of *Staphylococcus aureus* D48

*S. aureus* D48 (OD_600_ = 0.6) was treated with triple MIC of the bacteriocin ZFM216 solution at 37°C for 1 h. Untreated strains served as the control. After being washed with a phosphoric acid buffer (pH 7.0, 0.1 M), the thalli were mixed with 2.5% glutaraldehyde solution (4°C) for 12 h and then cleaned with the same buffer. After that, the sample was fixed with 1% osmic acid (SPI Supplies, United States) for 1 h and then rinsed with the same buffer again. The double-fixed bacteria were dehydrated with 30% to 100% ethanol (increasing by 10%) for 20 min each. The sample was dried by freezing and embedded with gold-palladium, and a Hitachi Su-8010 SEM was used for observation.

### Transmission electron microscopy of *Staphylococcus aureus* D48

The double-fixed sample prepared by the same method was dehydrated with 30%, 40%, 50%, 60%, 70%, 80%, 90% and 95% ethanol for 15 min. After being treated with pure ethanol for 25 min and with pure acetone (Sinopharm Group Chemical Reagent, China) for 25 min, the cells were placed in the embedding agent (v/v = 1:1 for 1 h) and acetone (v/v = 3:1 for 3 h) and then treated with embedding agent (SPI Supplies, United States) overnight. Then, the sample was heated to 70°C and this temperature was maintained for 12 h. The sample was cut into 80 nm sections and dyed with lead citrate (Sinopharm Group Chemical Reagent, China) and uranyl acetate solutions (SPI Supplies, United States) for 8 min each. Finally, photographs were taken with an electron microscope (Japan).

### Analysis of ATP levels

*S. aureus* D48 was cultured in 10 mL of LB liquid at 37°C to OD_600_ = 0.6 and centrifuged (5,000 rpm, 20 min) to collect the precipitates. Then, the precipitate was washed with 5 mM HEPES buffer (Sigma-Aldrich, United States) and redissolved to 5 mL. Fifty microlitres of a 10 mM glucose solution and 100 μL of cultured bacteria were mixed with bacteriocin solution at a final concentration of 1 × MIC. A 1% Triton X-100 solution acted as the positive control, and an equal volume of ultrapure water acted as the negative control. Eight samples were set in each group in different Eppendorf tubes. Every 5 min, one tube was centrifuged at 12,000 rpm for 2 min, and the supernatant fluid was discarded. Thirty microliters of ATP detection lysate and 120 μL of ATP detection reagent working solution were mixed and added to every tube, and the precipitate was resuspended. A black 96-well plate was used to contain the samples. Their fluorescence intensity was detected by a multifunctional micrometer.

### Analysis of the transmembrane electrical potential

The thallus of *S. aureus* D48 was obtained by the same method. Then the cells were washed with 5 mM fluorescent leakage buffer and resuspended to 5 mL. The excitation and emission wavelengths were set to 650 nm and 670 nm, respectively. The EX slit was 5 nm, the EM slit was 5 nm, and the scanning time was 15 min. A total of 20 μL of bacteria suspension and 3 μL of 0.1 mM probe dissc2 (Kumariya et al., 2019) were added to 2 mL of fluorescent leakage buffer in the fluorescent cuvette, mixed and detected. After the fluorescence value was stable, bacteriocin ZFM216 was added at a 1 × MIC concentration. The same volume of buffer and 5% Triton X-100 solution were added to the negative and positive controls.

### Analysis of conductivity

The probe of the conductivity meter was inserted into the *S. aureus* D48 culture, which was prepared by the same method and the initial conductivity was recorded. Bacteriocin ZFM216 was added at a 1 × MIC concentration. Equal volumes of HEPES buffer and 1% Triton X-100 solution were added to the negative and positive controls. The conductivity was recorded every minute until the value stabilized (Shanghai Precision Instrument).

## Results

### Bacteriocin purification

**Figure 1:**
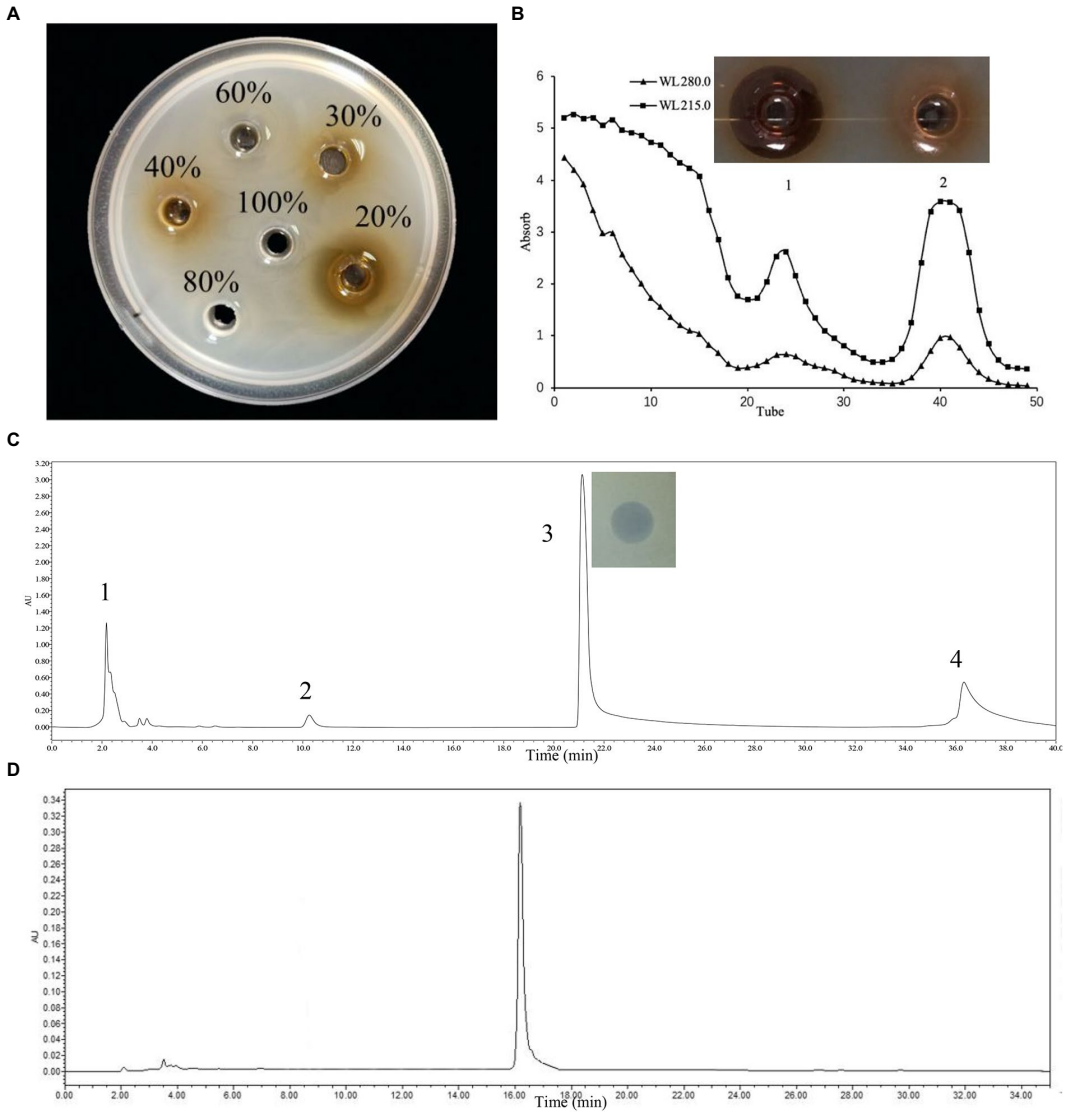
Three-step purification process of bacteriocin ZFM216 by chromatography column and antibacterial activity of absorbance peaks against *S. aureus* D48. **(A)** Macroporous resin. **(B)** Gel filtration. **(C)** Preparative PP-HPLC, peak 3 was the purified bacteriocin ZFM216. **(D)** Verify the purity of bacteriocin ZFM216 by analysis HPLC.

One-liter of *L. rhamnosus* ZFM216 supernatant was adsorbed by the XAD-16 macroporous resin. Under 20% ethanol, the eluent had good antibacterial activity (Figure 1A). The active protein was separated by gel chromatography. The absorbance of the eluent was detected at 280 nm and 215 nm. As it shown in Figure 1B, there were two peaks. Only the sample of peak 1 showed good antibacterial activity. After purification with Sephadex gel LH-20, the active part was purified by preparative RP-HPLC on a C18 column. As Figure 1C shows, four peaks eluted, and the component of the third peak had good antibacterial activity. Analytical HPLC was used for analyzing component 3 (purified bacteriocin ZFM216). Figure 1D shows that the monochromatic spectrum peak appeared at 16.34 min. Through the peak area normalization method, the purity of the peak was 97.3%, indicating that bacteriocin was well purified through the above methods. The concentration of protein was determined by a BCA protein kit, and the yield was 5.65 mg/L.

### Molecular mass of purified bacteriocin

**Figure 2:**
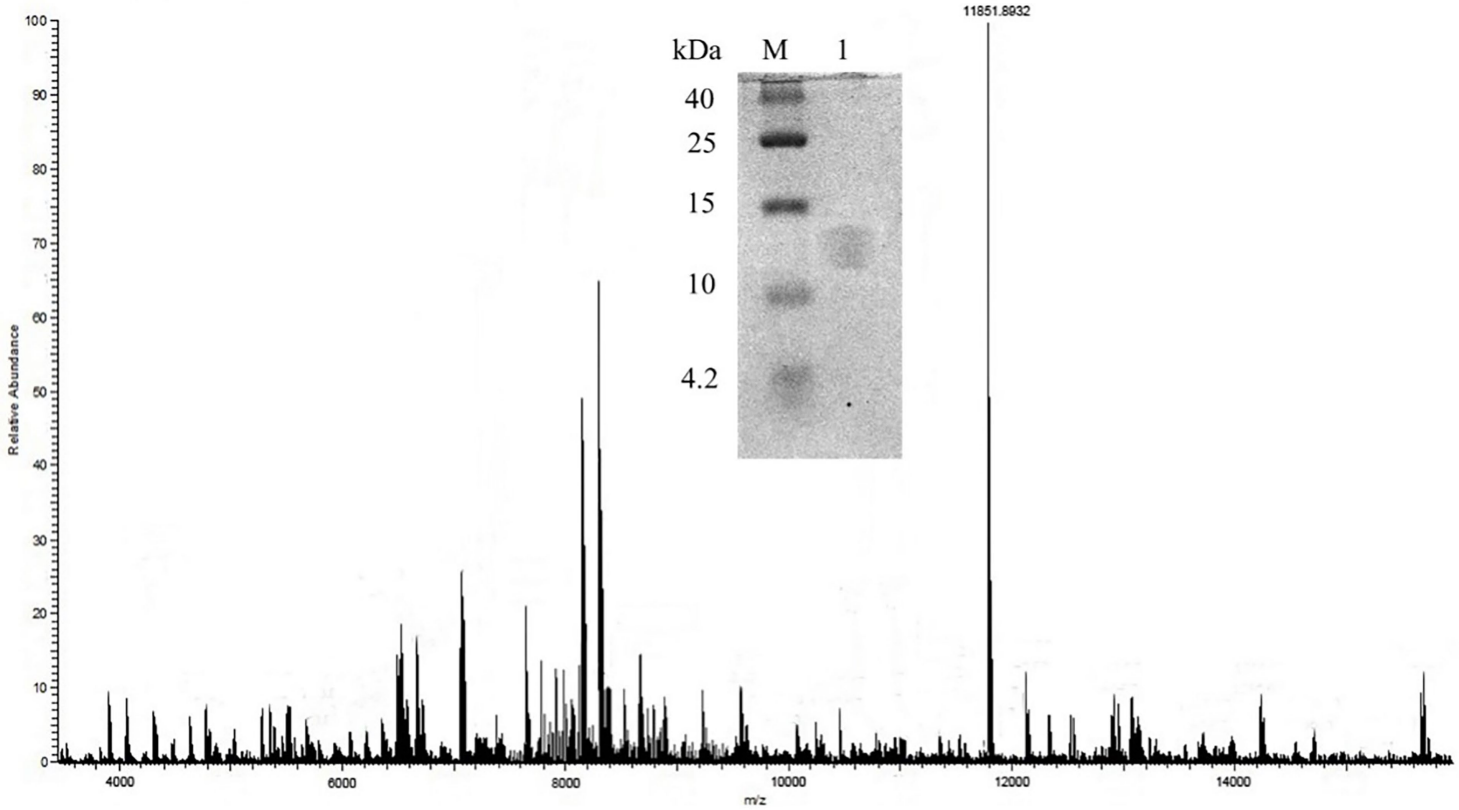
Molecule weight of bacteriocin ZFM216 by MALDI-TOF-MS and tricine-SDS-PAGE.

The molecular weight of the bacteriocin sample obtained by preparative RP-HPLC was estimated using tricine SDS-PAGE. As the result showed in Figure 2, the molecular mass of bacteriocin ZFM216 was between 10,000 Da and 15,000 Da. Through MALDI-TOF MS, the accurate molecular mass of this bacteriocin was determined 11851.9 Da.

### Antimicrobial spectrum and MIC

The bacteriostatic spectrum of bacteriocin ZFM216 was illustrated in Table 1. It had high antibacterial ability against most Gram-positive bacteria, such as *Staphylococcus aureus* and *Listeria monocytogenes* with MIC values 1.75 μM and 3.50 μM. At the same time, it also had antibacterial activity against Gram-negative bacteria such as *E. coli*, *Pseudomonas aeruginosa* and *Salmonella cholerae* with MIC values 1.75 μM, 3.50 μM, and 3.50 μM respectively. As the results show, this bacteriocin had broad-spectrum bacteriostatic activity.

**Table 1:** Antibacterial spectrum of bacteriocin ZFM216.

Strain name	Bacteriostatic circle diameter (mm)	MIC (μM)
*Staphylococcus aureus* D48	14.03 ± 0.29	1.75
*Staphylococcus warneri*	11.49 ± 0.69	7.00
*Micrococcus luteus* 10209	15.97 ± 0.18	0.875
*Staphylococcus carnosus* pCA 44	12.98 ± 0.14	3.50
*Staphylococcus carnosu*s pet 20	13.33 ± 0.64	3.50
*Listeria monocytogenes* LM1	12.90 ± 0.37	3.50
*Bacillus subtilis* BAS2	11.78 ± 0.31	7.00
*Escherichia coli* DH5α	14.91 ± 0.07	1.75
*Salmonella paratyphi-B* CMCC 50094	/	/
*Salmonella paratyphi-A* CMCC 50093	/	/
*Salmonella typhimurium* CMCC 50015	/	/
*Salmonella enterica subsp. arizonae* CMCC(B) 47001	11.95 ± 0.16	7.00
*Salmonella choleraesuis* ATCC 13312	12.89 ± 0.55	3.50
*Pseudomonas aeruginosa* ATCC 47085	12.88 ± 0.62	3.50

“/” was no inhibition.

### Stability against heat, pH and enzyme

**Figure 3:**
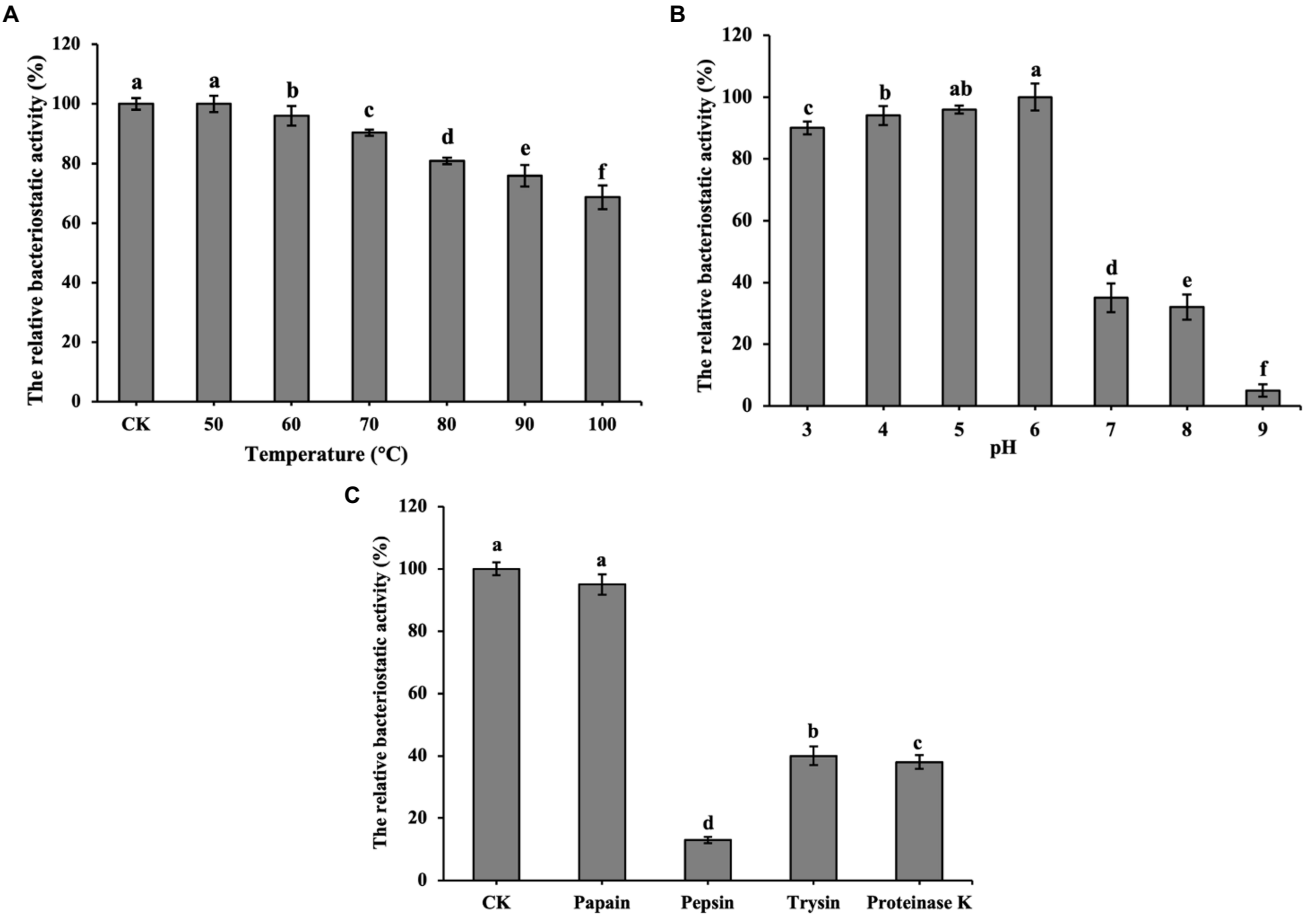
**(A)** Influence of temperatures on the antibacterial activity of bacteriocin ZFM216; **(B)** Influence of different pH on the antibacterial activity of bacteriocin ZFM216; **(C)** influence of different protease on the antibacterial activity of bacteriocin ZFM216 (different letters indicate significant differences in data).

Figure 3A shows that bacteriocin ZFM216 which maintained 81% antibacterial activity after treatment at 80°C and 69% activity after treatment at 100°C, was relatively heat stable. Figure 3B shows that bacteriocin ZFM216 had better antibacterial activity under weakly acidic conditions (between pH 3 and 6) than under alkaline conditions (between pH 7 and 9). Figure 3C shows that bacteriocin ZFM216 could be enzymatically broken down by pepsin, proteinase K, trypsin but insensitive to papain.

### Mode of mechanism of bacteriocin ZFM216 against *Staphylococcus aureus* D48

**Figure 4:**
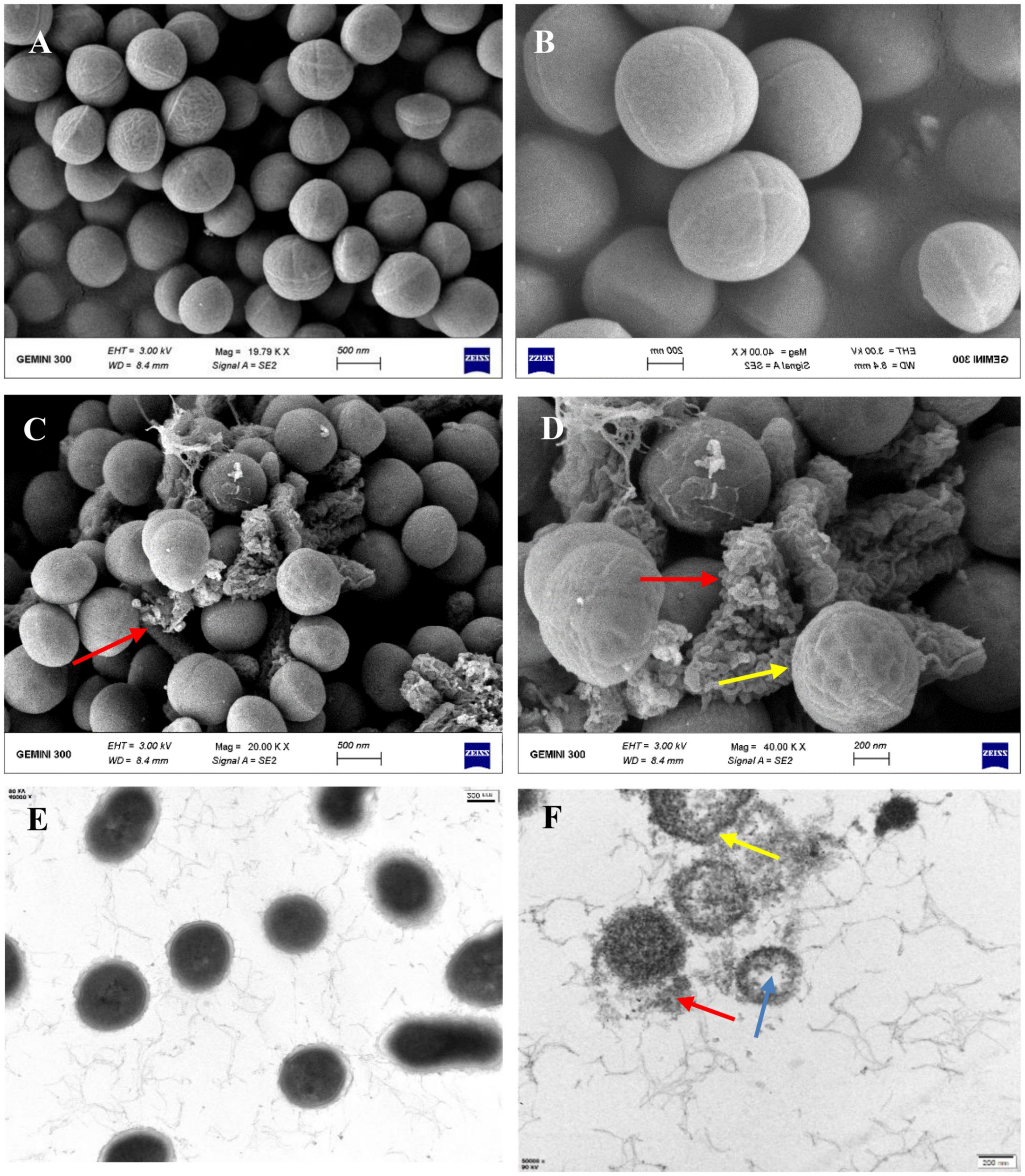
**(A,B)** The cell surface structure of untreated *S. aureus* D48 by SEM; **(C,D)** the characteristic of cell surface structure after treatment for 1 h by SEM (red arrows indicate broken cells; Yellow arrows indicate a wrinkled surface); **(E)** the cell surface structure of untreated S. aureus D48 by TEM; **(F)** the internal structural characteristics of cells after treatment for 1 h by TEM (blue arrows indicate intracellular cavities; yellow arrows indicate cell membrane holes; the red arrow indicates the outflow content).

To determine the antibacterial mode of bacteriocin ZFM216, the ultrastructural variation of *S. aureus* D48 cells after treatment with bacteriocin was observed by SEM and TEM. Figures 4A,B shows the surface of untreated *S. aureus* D48 cells, with complete cell structures and smooth and round membrane surfaces. Figures 4C,D shows the same cells treated with bacteriocin ZFM216, with wrinkled and damaged cell surfaces. Some cells were even completely dissolved. Untreated *S. aureus* D48 (Figure 4E) had a complete cell structure with a cell wall and membrane. The cytoplasm was evenly and densely distributed. However, as it shown in Figure 4F, the cell structure treated with bacteriocin ZFM216 was seriously destroyed, the membrane and wall of the cells were damaged to a certain extent, and the cytoplasm flowed out.

**Figure 5:**
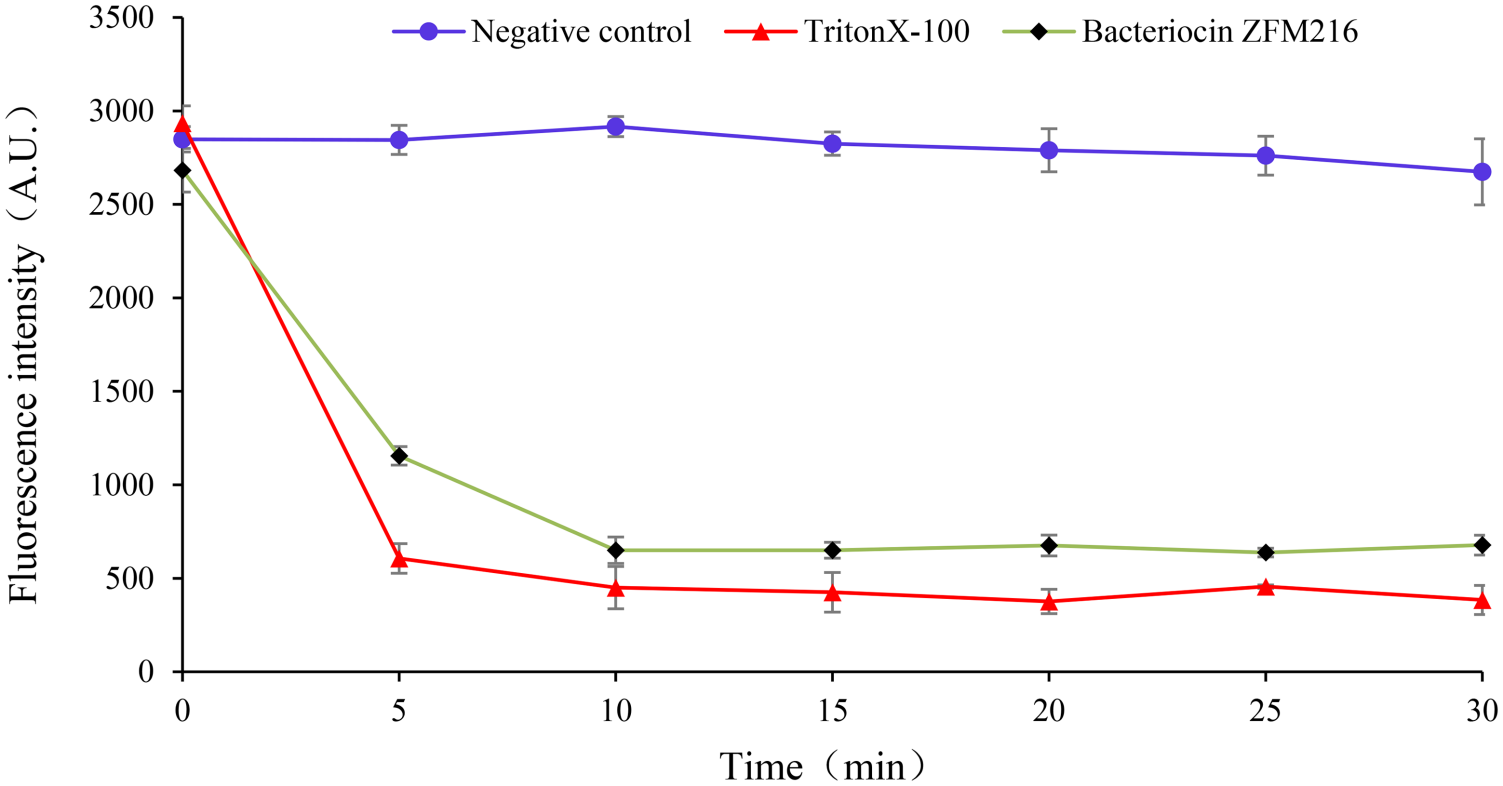
The effect of bacteriocin ZFM216 on the ATP level of *S. aureus* D48.

As it shown in Figure 5, similar to the result of the positive control group, the ATP level of *S. aureus* D48 treated with bacteriocin ZFM216 decreased rapidly, while the negative control remained unchanged. This bacteriocin damaged the surface of *S. aureus* D48 cells, which resulted in the dissipation of the proton driving force. Therefore, the cells were unable to synthesize ATP, and intracellular energy metabolism was disordered.

**Figure 6:**
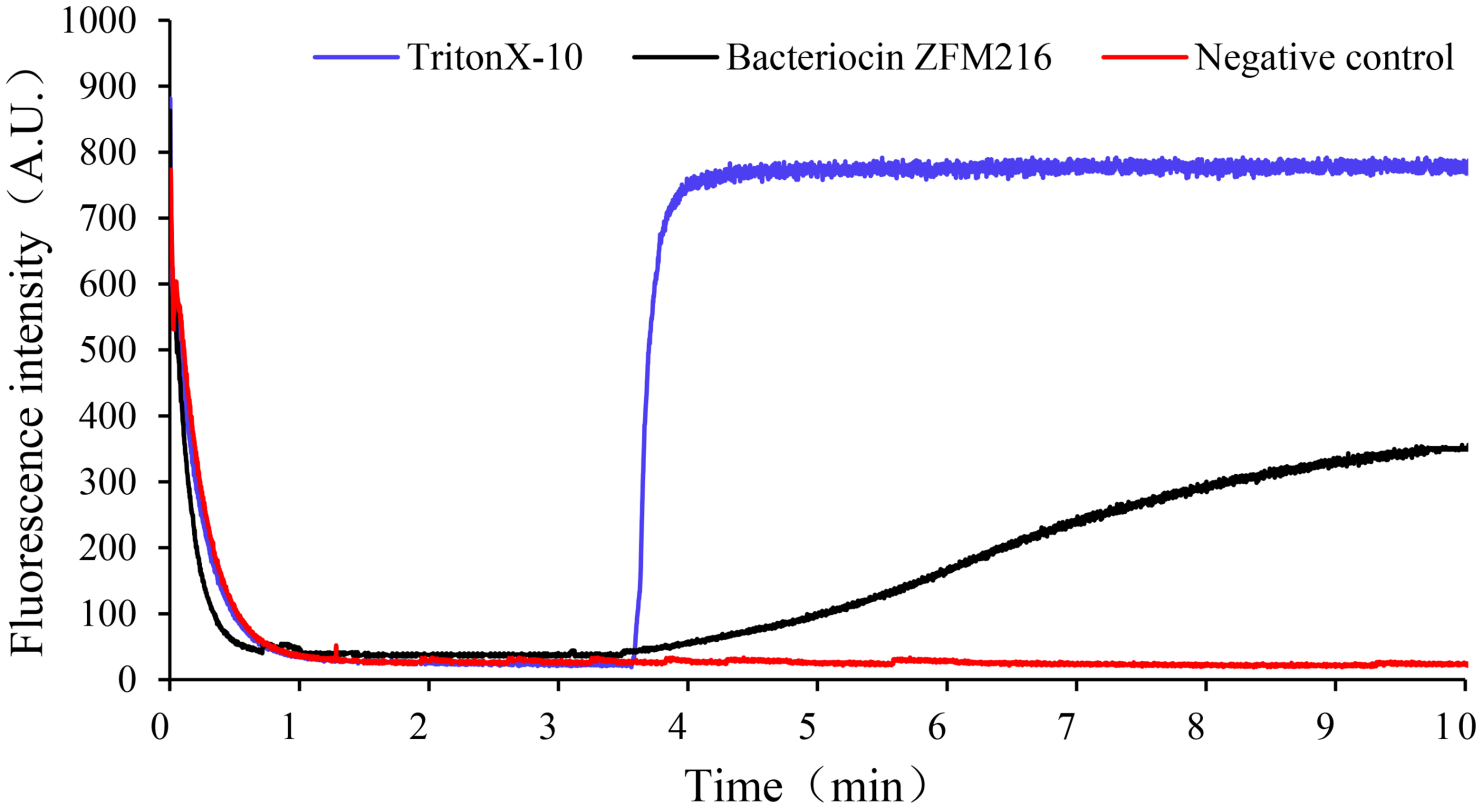
The effect of bacteriocin ZFM216 on the ΔΨ of indicator bacteria cell membrane.

Membrane potential refers to the potential difference across the living cell membrane. The destruction of sensitive bacterial cell membranes will cause potential difference dissipation. A fluorescent leakage test was performed to explore whether the bacteriocin could form membrane pores on *S. aureus* D48 and cause potential difference dissipation. As shown in Figure 6, the fluorescence intensity of indicator strains treated with bacteriocin ZFM216 increased. The fluorescence intensity of *S. aureus* D48 cells treated with Triton X-100 increased rapidly to the highest value, while that in the negative control remained unchanged. This result indicated that bacteriocin ZFM216 destroyed the cytomembrane of *S. aureus* D48.

**Figure 7:**
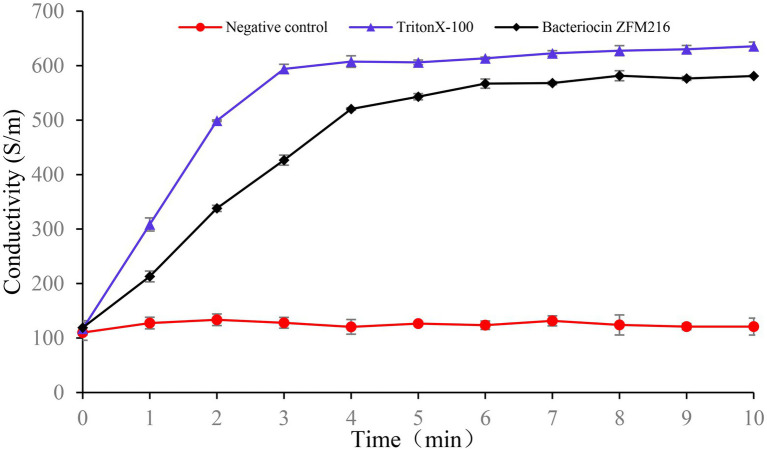
The effect of bacteriocin ZFM216 on the conductivity of indicator bacteria.

In the conductivity experiment, the results showed that after treatment with bacteriocin ZFM216, the conductivity of the *S. aureus* D48 suspension increased to the highest value within 5 min and remained stable, which was consistent with the trend of the positive control, while the ATP level of the negative control remained unchanged, as shown in Figure 7. This result indicated that bacteriocin ZFM216 destroyed the cytomembrane of *S. aureus* and caused electrolyte outflow.

## Discussion

*L. rhamnosus* is a species of LAB, which is frequently considered a beneficial organism and used as a probiotic. *L. rhamnosus* has been discovered to produce bacteriocin, such as acteriocin GP1 produced by *L. rhamnosus* GP1 (Sarika et al., 2019), rhamnosin A synthesized by *L. rhamnosus* strain 68, bacteriocin RC 20975 produced by *L. rhamnosus* CICC 20975 (Dimitrijević et al., 2009) and an antilisterial bacteriocin from *L. rhamnosus* CJNU 0519(Jeong and Moon, 2015). Because of their potent antibacterial activity against food spoilage and pathogenic bacteria, these kinds of bacteriocins could act as biopreservative or antibiotic alternatives. Various bacteriocin producing LAB have been found in fermented food (Parveen Rani et al., 2016; Therdtatha et al., 2016; Lv et al., 2017; Zhang et al., 2022), animal intestines (An et al., 2015), human feces (Maldonado-Barragán et al., 2016), soil and other resources. The antibacterial activity is mainly detected by spot-on-the-lawn, agar well diffusion (Yang and Chang, 2010) and drilling methods.

As an extracellular metabolite, the content of bacteriocin in CFS is relatively low (Halimi et al., 2010). Purification is the key step to obtain pure bacteriocin. The appropriate purification processes vary for bacteriocins with different structures and molecular masses. A bacteriocin from *L. rhamnosus* CJNU 0519 was partly purified by the acetone extraction method (Jeong and Moon, 2015). Acteriocin GP1 from *L. rhamnosus* GP1 was produced by the pH-adsorption method (Sarika et al., 2019). The purification of bacteriocin RC 20975 from *Lacticaseibacillus rhamnosus* CICC 20975 was conducted with ammonium sulfate precipitation followed closely using SP-Sepharose cation-exchange column and HPLC with a C18 reverse-phase column, and its molecular mass was 6502 Da. In this study, bacteriocin ZFM216 was obtained by a three-stage method consisting of the use of macroporous resin, gel chromatography and RP-HPLC. The purity rate of bacteriocin was 97.3%. The molecular weight varies largely between bacteriocins from LAB. Such as the bacteriocin named BAC-IB17 (10.7 kDa; Ansari et al., 2018), Gassericin E (5468.0 Da; Maldonado-Barragán et al., 2016), Plantaricin ZJ008 (1334.77 Da; Zhu et al., 2014) and so on. In most cases, the purified bacteriocin was identified by mass spectrometry (Pei et al., 2020; Qiao et al., 2020). Through these methods, the molecular mass of bacteriocin ZFM216 was identified as 11851.9 Da, which differs from previous bacteriocins reported by others. Thus, bacteriocin ZFM216 could be inferred as a novel bacteriocin except class I (<5 kDa) and class III (>30 kDa). It was thermostable and had only one activated peak on HPLC and one band by SDS-PAGE. Therefore, bacteriocin ZFM216 might be a class II bacteriocin but not belong to class IIb (dipeptide bacteriocin; Zhu et al., 2021).

Many bacteriocins produced from *L. rhamnosus* can inhibit various of bacteria. For example, bacteriocin RC 20975 can inhibit *Alicyclobacillus acidoterrestris*, *Bacillus subtilis*, *Lactobacillus acidophilus*, *Lactobacillus brevis*, *and Listeria innocua*. Similar to some other bacteriocins like bacteriocin-IB45 with broad spectrum of inhibition (Ansari et al., 2020; Ibrahim et al., 2020), bacteriocin ZFM216 has bacteriostatic effects on most Gram-positive bacteria, such as *L. monocytogenes* and *S. aureus*, and some Gram-negative bacteria, such as *E. coli, P. aeruginosa* and *S. cholerae*. It is noteworthy that *S. aureus* produces an array of toxins to promote disease. It is a representative food pathogenic bacterium causing food borne illness (Cheung et al., 2021). So effective control methods are urgently needed. For bacteriocin ZFM216, the MIC against *S. aureus* D48 was 1.75 μM. This provides a more effective inhibition than other bacteriocins, such as bacteriocin BMA, at 93.17 μM (Yi et al., 2016). Nisin is limited to only inhibiting Gram-positive bacteria. Therefore, we could believe that bacteriocin ZFM216 has strength and great potential in food preservation.

Bacteriocin ZFM216 was relatively heat-resistant and remained active at pH 3–6, allowing it to be applied in the food industry. In particular, its strong antibacterial activity in a weakly acidic environment means that it can be used in acidic food. Bacteriocin ZFM216 is sensitive to pepsin, trypsin and protease K. This characteristic of sensitivity to proteases could prevent their accumulation in the body and enhance their safety (Hemu et al., 2016). Bacteriocin ZFM216 is insensitive to papain. This is probably because the substrate non-specificity of papain for bacteriocin ZFM216 or the catalytic triad (Ser-His-Asp) of papain can’t act on bacteriocins (Tacias-Pascacio et al., 2021). A complete description requires a knowledge of both structure of papain and bacteriocin (Mccammon et al., 1976). Molecular dynamics and docking simulations could be carried out to explanation this phenomenon in the future (Nishiyama, 2011).

Some antibacterial modes of bacteriocins have been verified, such as inhibition of the cell wall, nucleic acids, protein synthesis and pore formation (Kumariya et al., 2019). The integrity of cell walls and membranes is crucial for the living cells (Cui et al., 2021). Cell permeabilization and pore formation are the main bacteriostatic modes of bacteriocins (Moll et al., 1999). Different methods must be used to explain the antibacterial mechanism from different aspects. In SEM, a high-energy electron beam is used to scan the test sample and observe the micro morphology of the sample. In TEM, some internal structures of cells can be observed. These two methods were used by Li et al. (Zhang et al., 2016) to infer that the mode-of-action of the two-peptide bacteriocin plnEF on sensitive strains causes cell membrane damage. Bacteriocin BM1029, which can cause pore formation on its indicator, was detected by TEM and SEM (Yan et al., 2021). The influence of bacteriocin (BAC-IB17) on membrane integrity of methicillin-resistant *S. aureus* was also confirmed by SEM (Ansari et al., 2020). Plantaricin JY22 can improve ATP levels in tested bacteria (Lv et al., 2018). The effect of bacteriocins on the membrane permeability of susceptible bacteria can be explored using fluorescent probes such as DISC2 (Kumariya et al., 2019), the sodium ion fluorescent probe SBFI AM, immunoglobulins and calcein. Through a leakage assay, a class IIB bacteriocin plnJK, was discovered to cause the following changes. The increasing of the indicator cytomembrane permeability, leading to the leakage of contents, a decrease in cell membrane potential difference and finally cell death (Ladokhin et al., 1997). By detecting extracellular conductivity changes in sensitive bacteria, Qiao found that Enterocin TJUQ1 could alter the ion channels in the indicator cell membrane (Qiao et al., 2020).

Currently, we rarely find evidence of the mode-of-action of bacteriocin against *S.aureus*. This research provides the antibacterial mode-of-action bacteriocin ZFM216 on *S. aureus* D48 to provide a theoretical basis for its application as a technique in biopreservation. The results of electron microscopy showed that the cell structure of *S. aureus* D48 treated with bacteriocin ZFM216 was seriously deformed, the cell membrane of *S. aureus* D48 showed obvious shrinkage, some holes appeared on the membrane, and the intracellular electrolyte leaked out of the cell. Such morphological changes suggest that the cellular membrane might be the target. Bacteriocin ZFM216 caused a decrease in cellular ATP levels, a dramatic increase in electrical conductivity, and a transient decrease in the transmembrane potential difference (ΔΨ) of *S. aureus* D48. All the results above confirmed that the antimicrobial mode-of-action of bacteriocin ZFM216 is membrane disruption. This bacteriocin leads to the rupture of the cytomembrane of indicator strains, the inhibition of intracellular ATP synthesis, and the leakage of intracellular electrolyte to the outside of cells, leading to the death of the indicator. This finding will provide valuable information for antibacterial agents against *S. aureus* (Zhou et al., 2020; Pu et al., 2022).

In further studies, the structural gene, amino acid sequence, structure of bacteriocin ZFM216 and more details about the antibacterial mode will be addressed. Moreover, before application, the safety of this bacteriocin should be tested.

## Data availability statement

The raw data supporting the conclusions of this article will be made available by the authors, without undue reservation.

## Author contributions

QG, PL and YS conceived and designed the study. DW, MD, and QZ completed the experiment. DW conducted analysis the results and finished the paper. All authors contributed to the article and approved the submitted version.

## Funding

This project was funded by the Chinese Academy of Engineering Academy-Locality Cooperation Project (No. 2019-ZJ-JS-02), the Key Research and Development Program of Zhejiang Province (No. 2020C04002) and the Joint Funds of the National Natural Science Foundation of China (No. U20A2066).

## Conflict of interest

The authors declare that the research was conducted in the absence of any commercial or financial relationships that could be construed as a potential conflict of interest.

## Publisher’s note

All claims expressed in this article are solely those of the authors and do not necessarily represent those of their affiliated organizations, or those of the publisher, the editors and the reviewers. Any product that may be evaluated in this article, or claim that may be made by its manufacturer, is not guaranteed or endorsed by the publisher.
